# Availability of patient-centered cancer support services: A statewide survey of cancer centers

**DOI:** 10.1371/journal.pone.0194649

**Published:** 2018-03-27

**Authors:** Kathleen B. Cartmell, Katherine R. Sterba, Kim Pickett, Jane Zapka, Anthony J. Alberg, Amit J. Sood, Nestor F. Esnaola

**Affiliations:** 1 College of Nursing, Medical University of South Carolina, Charleston, SC, United States of America; 2 Hollings Cancer Center, Medical University of South Carolina, Charleston, SC, United States of America; 3 Department of Public Health Sciences, Medical University of South Carolina, Charleston, SC, United States of America; 4 Arnold School of Public Health, University of South Carolina, Columbia, SC, United States of America; 5 Department of Surgery, Fox Chase Cancer Center, Philadelphia, PA, United States of America; TNO, NETHERLANDS

## Abstract

The Institute of Medicine recommended in their landmark report “From Cancer Patient to Cancer Survivor: Lost in Transition” that services to meet the needs of cancer patients should extend beyond physical health issues to include functional and psychosocial consequences of cancer. However, no systems exist in the US to support state-level data collection on availability of support services for cancer patients. Developing a mechanism to systematically collect these data and document service availability is essential for guiding comprehensive cancer control planning efforts. This study was carried out to develop a protocol for implementing a statewide survey of all Commission on Cancer (CoC) accredited cancer centers in South Carolina and to implement the survey to examine availability of patient support services within the state. We conducted a cross-sectional survey of CoC-certified cancer centers in South Carolina. An administrator at each center completed a survey on availability of five services: 1) patient navigation; 2) distress screening; 3) genetic risk assessment and counseling, 4) survivorship care planning; and 5) palliative care. Completed surveys were received from 16 of 17 eligible centers (94%). Of the 16 centers, 44% reported providing patient navigation; 31% reported conducting distress screening; and 44% reported providing genetic risk assessment and counseling. Over 85% of centers reported having an active palliative care program, palliative care providers and a hospice program, but fewer had palliative outpatient services (27%), palliative inpatient beds (50%) or inpatient consultation teams (31%). This was a small, yet systematic survey in one state. This study demonstrated a practical method for successfully monitoring statewide availability of cancer patient support services, including identifying service gaps.

## Introduction

As of 2016 the US had more than 15.5 million cancer survivors [1]. With an aging population and medical advances that enable more effective cancer screening and treatment, estimates indicate a 36% increase in the number of cancer survivors to 19 million by 2024 [1]. In recognition of the needs of the growing population of cancer survivors, the Institute of Medicine (IOM) published two landmark reports “From Cancer Patient to Cancer Survivor: Lost in Transition” [2] and “Cancer Care for the Whole Patient: Meeting Psychosocial Health Needs [3].” These reports have raised awareness that the needs of cancer patients extend beyond physical health issues into the functional and psychosocial consequences of cancer and its treatment.

To help translate recommendations from the IOM reports into tangible support services that can benefit those living with cancer, the American College of Surgeon’s Commission on Cancer (CoC) has added new cancer center accreditation standards. These program standards are described in their 2012 cancer program standards [4]. Genetic risk assessment and counseling services and palliative care were both added as new program standards by the CoC in 2012 [4]. They also introduced three new program standards in their 2012 program standards document for phase-in by 2015 [4]. Patient navigation and psychosocial distress screening became active new standards in January 2015 [5]. Survivorship care planning was also scheduled to be added as a new program standard by January 2015, but due to the scope of work that will be required to develop these services, cancer centers will now have until the end of 2018 to fully phase in this service [5]. Together, these services are designed to improve the quality and continuity of cancer care, but little is known about the extent to which these services have been implemented.

These services are a priority for cancer control planning efforts at the state level across the US. Every state and territory in the US receives funding from the Centers for Disease Control and Prevention (CDC) to create a comprehensive state cancer plan for decreasing the burden of cancer. State cancer alliances generally include partners such as public health agencies, cancer centers, academic institutions, cancer patients and survivors and advocacy organizations such as the American Cancer Society and the Komen Foundation. To inform state cancer control planning efforts, these cancer control partners require state-level data about the availability of patient support services.

Unfortunately, despite the IOM recommendation for greater emphasis on helping cancer patients navigate the cancer care experience and the presence of state cancer control coalitions across the country, there are currently no statewide systems for measuring the availability of patient support services. The US Health Information National Trends Survey [6] collects data on the availability of some patient support services such as survivorship care planning, but these data are not collected at a state level [7,8]. As part of the CDC Behavioral Risk Factor Surveillance Survey, there are also a few cancer survivorship related-questions that can be added by states as supplemental questions, but due to the topical breadth of the survey and the cost of adding supplemental questions, these questions are only sporadically included on state surveys. Systematic data collection strategies are urgently needed in order to track progress in making essential patient care services available for cancer patients. To address this gap in the evidence, the current project was undertaken to develop and implement an easy-to-administer cross-sectional survey to document the availability of five key CoC accreditation standards for cancer centers: 1) patient navigation, 2) distress screening, 3) genetic risk assessment and counseling, 4) survivorship care planning, and 5) palliative care.

## Methods

A cross-sectional on-line survey protocol was developed and carried out in February-April 2012 among CoC accredited cancer centers in South Carolina. Twenty-one ACoS-CoC cancer centers in the state were initially identified from the national CoC organization. For each cancer center, we obtained a list of the center team members who were responsible for oversight and coordination of CoC program standards. Team members typically included the CoC program coordinator, liaison physician and CoC program chair. Of the 21 centers, two were excluded from the survey as they were no longer CoC certified. Three centers were part of a larger health care system and were included as a single cancer center system. In total, seventeen cancer center systems met inclusion criteria for survey participation. This project was reviewed by the Medical University of South Carolina Institutional Review Board and deemed to not constitute human research.

The survey was designed to include questions about the availability of five key services: 1) patient navigation; 2) distress screening; 3) genetic risk assessment and counseling, 4) survivorship care planning; and 5) palliative care. At the time of this study, there were no validated items to measure these implementation questions. Thus, items were developed with an emphasis on content validity based on review and adaptation of topical content from published literature related to each topic. Survey questions were designed to parallel the new program standards described in the 2012 CoC accreditation standards (genetic risk assessment and counseling and palliative care as a 2012 requirement; patient navigation, distress screening and survivorship care planning as a requirement for planned phase-in) [4]. The instrument was reviewed by three professionals with expertise in oncology, patient-centered support services and survey methodology, which resulted in initial instrument refinement. The instrument also underwent cognitive pretesting with three cancer center staff from across the state who were involved with implementation of the CoC accreditation standards, which resulted in final changes to the instrument.

To conduct the survey, we first contacted each cancer center to identify the names and contact information for each member of the CoC team at each center. We then sent a joint email to the CoC team at each cancer center to ask that they designate one person to complete the survey. The email message included a cover letter from state leaders of the cancer alliance, the CoC and the American Cancer Society, and it contained a link to fill out the survey online.

### Patient navigation

Patient navigation questions queried key parameters described in existing navigation literature reviews [9–11]. The CoC Standard 3.1 requires the cancer committee at each cancer center to provide navigation services either on site or by referral or in partnership with local or national organizations [5]. Survey questions assessed the level of availability of this service to provide individualized assistance to patients to access and understand the care they need (*regularly available*, *limited availability*, *lack of availability*), presence of navigation services by tumor type (*blood/lymph node*, *bone marrow*, *brain/spinal*, *breast*, *gastrointestinal*, *genitourinary*, *gynecologic*, *head/neck*, *thoracic*, *melanoma/skin*, *pediatric*, *sarcoma*, *other*), navigators’ professional qualifications (*lay navigator*, *professional navigator trained in nursing or social work*, *other*), and points in care when assistance was provided (*screening*, *diagnosis*, *treatment*, *post-treatment*, *other*).

### Distress screening

Distress screening questions were based on a review of common distress screening intervals and instruments reported in the literature [11]. The CoC Standard 3.2 requires the cancer committee at each cancer center to recognize and address the psychosocial distress of persons with cancer [5]. Survey questions focused on the frequency of assessment of distress screening (*routinely conducted at cancer center*, *not routinely conducted at cancer center*, *but patients referred as needed for psychological services*, *psychosocial assessment and referral is not a part of routine care at our cancer center*), timing for distress screening across the cancer continuum (*initial visit*, *diagnosis*, *treatment initiation*, *after treatment completion*, *long-term follow up*, *recurrence*, *upon recognition that disease is incurable*, *end of life discussion*, *psychosocial assessment not performed*, *other*), and distress screening tool utilized (*NCCN thermometer*, *FACIT Scales*, *Hospital Anxiety and Depression Scale*, *Center for Epidemiological Studies Depression Scale*, *Brief Symptom Inventory*, *Profile of Moods*, *Zung Self-Report Depression Scale*, *no psychosocial assessment tools*, *other)*.

### Genetic risk services

Genetic risk assessment and counseling questions were adapted from CoC 2012 standard audit statements [4]. The CoC Standard 2.3 requires the cancer committee at each cancer center to provide risk assessment and genetic testing, either on site or by referral, by a qualified genetics professional [5]. Survey questions focused on whether genetic cancer risk assessment and counseling was obtained by a qualified professional and the availability. Responses included: Yes, *systematically provided; Not systematically provided*, *but with limited availability to some patients; No*, *not generally provided*.

### Survivorship care plans

Survivorship care plan questions reflected a review of common survivorship program parameters. The CoC Standard 3.3 requires, by the end of 2017, the cancer committee at each cancer center to develop and implement a process to provide a comprehensive treatment summary and follow up plan to ≥ 50% of eligible patients who have completed treatment [5]. Survey questions focused on assessment of information patients received as part of a survivorship care plans. Categories assessed included information about diagnosis, patient treatment details, appropriate schedule for follow-up visits or tests, potential treatment effects, advice on important lifestyle issues such as physical activity, smoking and diet, symptoms to watch for, and listing of support resources. Survey responses reflected the frequency of service availability as *never*, *rarely*, *sometimes*, *very often*, and *always*. A survey item assessed the format of survivor care plan templates employed at each cancer center (*NCCN Survivor Care Plan*, *Journey Forward*, *LiveStrong Care Plan*, *ASCO Treatment Plan/Summary*, *cancer center-specific plan*, *other*, *or lack of any plan*). Another survey item inquired if the cancer center had a staff person designated to work specifically on cancer survivorship care.

### Palliative care

Palliative care questions adapted CoC 2012 audit statements and reflected a review of essential palliative care service components that have been identified in the literature. The CoC Standard 2.4 requires palliative care services provided either on site or by referral [5]. Questions focused on availability of patient care services. Categories assessed if each cancer center currently had an active palliative care program, at least one palliative care physician, at least one palliative care nurse, an inpatient consultation team, outpatient referral availability, dedicated palliative care beds, and a hospice program. Responses were categorized as *available on site*, *available by referral*, and *unavailable*.

## Results

Completed surveys were received from 16 of 17 eligible centers (94%). Among the 16 cancer centers that participated in the survey, 56% (n = 9) were located in urban settings, with the remaining 44% (n = 7) in suburban or rural settings. In terms of type of cancer center, 13% (n = 2) were academic comprehensive cancer programs, 38% (n = 6) were comprehensive community cancer programs, 31% (n = 5) were community cancer programs, 13% (n = 2) were VA cancer programs and 6% (n = 1) was an integrated network cancer program. The single cancer center that did not agree to participate in the survey was a mid-sized community cancer program located in an urban setting. Below are the survey results from the 16 participating cancer centers for each of the five patient services. See S1 and S2 Files for the data dictionary and dataset on which the current analysis is based.

### Patient navigation

Details about the patient navigation programs are described in **Table 1**. Forty percent (n = 7) of cancer centers reported that navigation is regularly available, while 44% (n = 7) and 13% (n = 2) report that these services are available to some patients, and completely unavailable to others, respectively. Navigation programs were most commonly staffed by professional nurse navigators (75%) and the majority provided assistance across the cancer care continuum. Patient navigation was most commonly available to patients with breast (63%), thoracic (44%) and gastro-intestinal (44%) cancers. Few programs provided patient navigation services for patients with skin cancer, hematopoietic malignancies, sarcomas, gynecological or pediatric cancers.

**Table 1:** Characteristics of patient navigation, distress screening and genetic risk assessment/counseling at CoC cancer centers in South Carolina, 2012.

**Patient Navigation (n = 16)**		
**Survey Item**	**Categories**	**%**
Patient navigation service	Regularly available	44%
	Available for some patients	44%
	Not available	13%
Cancer types serd*	Breast	63%
	Thoracic	44%
	Gastro-intestinal	44%
	Genitourinary	38%
	Head & neck	38%
	Skin	19%
	Brain/spinal cord	19%
	Bone Marrow	19%
	Blood/lymph	13%
	Sarcoma	13%
	Gynecological	6%
	Pediatric	6%
Type of navigator	Professional navigator	75%
	Lay navigator	25%
Points of assistance*	Screening	56%
	Diagnostics	75%
	Treatment	81%
	Post-Treatment	69%
**Distress Screening (n = 16)**		
**Survey Item**	**Categories**	**%**
Distress screening	Routinely conducted	31%
	Not routine/referred as needed	56%
	Not conducted	13%
Point of distress screening*	Initial visit	44%
	At diagnosis	19%
	Start of treatment	25%
	End of treatment	13%
	Long term follow-up	13%
	Recurrence/incurable	19%
	End of life discussion	25%
Distress screening tool	NCCN Thermometer	25%
	Profile of Moods	6%
	Reported no tool, unknown or informal assessment	69%
**Genetic Risk Assessment and Counseling Services (n = 16)**		
**Survey Item**	**Categories**	**%**
Genetic cancer risk assessment for patients, either onsite or by referral, by a qualified professional	Yes, systematically provided across cancer center	38%
	Not systematically provided across cancer center; but available to some patients	44%
	No, not generally provided	19%
Genetic counseling services, either onsite or by referral, by a qualified professional	Yes, systematically provided across cancer center	44%
	Not systematically provided across cancer center, but available to some patients	38%
	No, not generally provided	19%

* Percentages will not sum to 100% because participants could check more than one answer for these items

### Psychosocial distress screening

Details about the psychosocial distress screening programs are described in **Table 1**. At centers that provided distress screening, it was most commonly performed upon initial visit (44%) and was less likely to be performed over time at subsequent intervals such as time of diagnosis (19%), treatment initiation (25%), treatment completion (13%), long-term follow-up (13%), recurrence (19%), and during end of life (25%) discussions. Most commonly, centers used informal assessment techniques (69%) rather than validated tools to assess distress.

### Genetic risk assessment and counseling services

Details about the genetic risk assessment and counseling services are described in **Table 1.** Thirty eight percent of centers reported that they systematically provide genetic cancer risk assessment for patients, 44% reported that they do not systematically provide these services, but that they are available to some patients, and 19% reported that these services are not generally provided. In terms of genetic counseling services, 44% of centers reported that these services are systematically provided across the center, 38% reported that they do not systematically provide these services across the center, but that they are available to some patients; and 19% reported that these services are not generally provided.

### Survivorship care plans

Details about the availability of survivorship care planning services are shown in **Table 2**. With respect to survivor care plans, the prevalence of “very often” or “always” responses to providing written information to patients were 40% for diagnosis, 40% for treatment, and 57% for follow-up care schedule (57%). The prevalence was similar for the provision of written information about long-term treatment effects, lifestyle modification advice, and support resources (40%). In terms of survivorship care plan templates, most (69%) lacked a formal cancer plan template. Some cancer centers reported using the following templates: Journey Forward (13%), Live Strong Care Plan (6%), NCCN Survivorship Care Plan (6%), Star Survivorship (6%) and a cancer center-specific developed care plan (6%). Only 16% of centers had a staff-person designated to work on survivorship care for their patients.

**Table 2:** Availability of survivorship care planning services in CoC cancer centers in South Carolina, 2012.

Extent to which patients receive information in written care plan: *	Never	Rarely	Sometimes	Very Often	Always
Patient diagnosis information (n = 15)	47%	0%	13%	20%	20%
Patient treatment details (n = 15)	53%	0%	7%	20%	20%
Appropriate schedule for follow-up visits/tests (n = 14)	36%	0%	7%	21%	36%
Potential long-term and late treatment effects (n = 15)	33%	7%	20%	20%	20%
Advice on important lifestyle issues (ie. physical activity, smoking and diet) (n = 15)	33%	0%	27%	20%	20%
Symptoms to watch (n = 15)	33%	7%	7%	33%	20%
List of support resources (n = 15)	33%	0%	27%	20%	20%

*Not all cancer centers responded to every item on the survey. The number of centers who responded to each item is provided.

### Palliative care

Details about the availability of palliative care services are described in **Table 3**. Fifty six percent (n = 9) of cancer centers reported having an active palliative care program available on site, while 38% (n = 6) and 6% (n = 1) reported palliative care availability through referral and lack of this service, respectively. Forty four to fifty six percent of centers (n = 7–9) reported having a palliative care physician, nurse and inpatient consultation team available on site, whereas 25–31% (n = 4–5) and 13–31% (n = 2–5) offered this service by referral or lacked this service, respectively.

**Table 3:** Availability of palliative care services at CoC cancer centers in South Carolina, 2012.

Availability of Palliative Care Resources*	Not Available	Available by Referral	Available on Site
Currently active palliative care program (n = 16)	6%	38%	56%
At least one palliative care physician (n = 16)	13%	31%	56%
At least one palliative care nurse (n = 16)	13%	38%	50%
Inpatient consultation team (n = 16)	31%	25%	44%
Outpatient clinic (n = 15)	73%	7%	20%
Dedicated palliative care beds (n = 16)	50%	6%	44%
Hospice program (n = 15)	7%	53%	40%

*Not all cancer centers responded to every item on the survey. The number of centers who responded to each item is provided.

### Overall service availability

Fifty-six percent of centers had an active, on-site palliative care program. With the data obtained, we calculated the number of the 5 services at each center, defined as: 1) patient navigation, regularly available; 2) distress screening routinely conducted; 3) survivorship care plans, all components included; 4) palliative care, 4/7 services available; and 5) genetic risk assessment and counseling, both systematically provided. Six percent of cancer centers (n = 1) offered all five services, 12% (n = 2) offered four services, 12% (n = 2) offered three services, 25% (n = 4) offered two services, and 44% (n = 7) offered one service.

## Discussion

A survey instrument was developed and administered to assess cancer patient support services at all CoC cancer centers in a single state. A simple and feasible data collection instrument and data collection protocol was developed that enables statewide characterization of availability and gaps in patient cancer support services. This provides a low resource strategy to generate valuable statewide tracking data to inform cancer control planning efforts.

The survey results identified considerable potential to expand support services for cancer patients and survivors across the state. Specifically, only 12% of cancer centers (n = 2) reported systematically offering three or more of the five services that were evaluated, with only 6% reporting systematically offering all five services (n = 1). Of the five services, the only service that was reported to be regularly provided by over half of cancer centers was palliative care, a service for which the CoC accreditation standard was put in place earlier in 2012. Of the remaining four services, 44% of cancer centers reported systematically providing patient navigation; 38% and 44% of cancer centers respectively provided genetic risk assessment and counseling; 33% provided distress screening; and 20–36% always provided various components of survivorship care planning.

Palliative care initiatives have been growing over the past two decades, as integration of oncology and palliative care is shown to be beneficial in many studies [12]. Most cancer centers in our survey had service components such as an active palliative care program, a palliative care nurse, and/or physician and a hospice program either available onsite or by referral. This finding suggests that some level of basic palliative care is available to patients across the state. However, the lack of a hospice outpatient program, dedicated palliative care beds and an inpatient consultation team at many centers suggests that additional components are needed in many areas across the state to be able to provide comprehensive palliative services. For example, palliative care beds and an inpatient consultation team would be needed to promote transition of patients from curative to palliative care to enhance comfort and support earlier in their illness. Similarly, hospice outpatient programs would be needed to meet the needs of terminally ill patients in their home environment, perhaps after a transition from inpatient palliative care [13]. Together these findings pinpoint clear areas for improvement to support holistic and streamlined systems for delivery of palliative care.

Patient navigation is a strategy that has received considerable attention over the last two decades, particularly as it applies to reducing health disparities [9,14]. However the components and services across patient navigation programs vary greatly [15]. Most cancer centers in our survey reported that patient navigation is regularly provided (44% of centers) or available for some patients (44% of centers). This finding demonstrates that there is at least a minimal level of navigation infrastructure in cancer centers across the state. Additionally, substantial differences in the availability of patient navigation across types of cancers were identified, with 63% of centers reporting having these services for breast cancer patients, 44% for gastro-intestinal and thoracic cancers, and less than 20% for more rare cancers. It is not surprising that patient navigation is most commonly provided for breast cancer patients, given that navigation in cancer care evolved as a service to improve diagnosis and follow up of women with an abnormal breast screening result [16, 17]. Most cancer centers reported that their patient navigators were nurses or social workers by training, with only a few centers reporting the use of lay navigators. A number of research studies have demonstrated that lay navigators can be useful to expand the reach of busy clinical staff [18]. Specifically lay navigators may be well suited to carry out non-clinical tasks such as helping patients to overcome logistical barriers to care (e.g. transportation, housing), filling out paperwork and scheduling and reminding patients about upcoming appointments and adherence to follow-up procedures and lifestyle changes [10]. The study provided evidence that patient navigation services are fairly well-integrated across cancer centers in the state, but that further expansion is needed to support patients with additional types of cancers.

In our study, distress screening was only routinely conducted among 31% of cancer centers. These results mirror those from a survey of cancer center professionals attending an educational conference, which reported that only 41% of participants reported that their institutions had begun distress screening at all [19]. While distress screening in our study was most commonly performed at the initial visit (44%), it was less likely at the time of diagnosis (19%) and at initiation of end of life care (25%). Screening patients for distress can provide specific information at key intervals of care about what services may be beneficial for patients. In a study that evaluated implementation of distress screening in cancer centers, clinical team members reported uncertainty about which medical encounters represent the pivotal visits when distress screening would be most beneficial [19]. In terms of screening tools, our study documented that 69% of cancer centers were not using a tool for distress screening. This finding is corroborated by results from a recent survey of cancer centers in Georgia, which found that 60% of cancer centers reported never or rarely using a psychosocial assessment tool for patients in their cancer center [20]. Together, these findings provide evidence that considerable work is still needed to achieve routine distress screening for all cancer patients.

Significant potential and need exists in terms of delivery of survivorship care plans. In our survey, cancer centers reported that they frequently (“very often” or “always”) provided written information to their patients about their diagnosis (40%), treatment (40%), and follow-up care schedule (57%). Similar levels of endorsement were seen for the provision of written information about long-term treatment effects, lifestyle modification advice, and support resources (40%). These findings are consistent with other studies, which have reported that less than half of cancer centers or oncologists provide survivorship care plans to their cancer survivors [20–22]. The breadth of survivorship care plans, which are designed to provide patients with information about their diagnosis, treatment, follow up schedule, potential long-term treatment effects, lifestyle modification and support resources, translates into collection of extensive information across various data sources to provide patients with complete, accurate and useful information. Cancer centers are clearly struggling to provide this service, as evidenced by recent delays in the dates by which centers must adhere to this standard [5].

Strengths and limitations of the current study should be acknowledged. A substantial strength of the survey was that 16 of the 17 (94%) of cancer centers in the state completed the survey, which helps to ensure generalizability of the survey results across the state. Another strength is the partnership in sponsoring the survey, which included the Commission on Cancer, the South Carolina Cancer Alliance and the American Cancer Society. A limitation of the survey though was that data was obtained from only one informant and no reliability or validation steps were undertaken. However, the individuals who completed each survey were the individuals at each cancer center (usually the CoC coordinator at each site) whose role was directly related to implementation of the new CoC standards. These individuals would likely be the most knowledgeable individuals at each cancer center to report on availability of these CoC standards of service. While validation of the survey was limited to review by three experts for survey content and structure and pretesting among three cancer center staff, the survey was built upon the CoC accreditation standards that had undergone substantial peer review, and at the time there was no existing instrument available to evaluate the implementation outcomes evaluated in our study. A second limitation is that this survey was carried out in 2012, and it is likely that progress has been made since that time among cancer centers towards implementation of the services assessed in our survey.

In summary, the new CoC accreditation standards provide an opportunity for cancer centers and their state cancer control planning partners to develop and maintain these important services. To inform state cancer control planning efforts, cancer control partners will require access to statewide information about what services are available for cancer patients and survivors in their state. The current study provides a simple, feasible, low resource approach to generate valuable data on this topic.

## Supporting information

S1 File Data dictionary. (DOCX)

S2 File Dataset. (XLSX)
