# Supplementary material for: Availability of patient-centered cancer support services: A statewide survey of cancer centers
Source: PLoS One. 2018 Mar 27;13(3):e0194649. doi: 10.1371/journal.pone.0194649 (PMC5870953; doi:10.1371/journal.pone.0194649)
Supplement: S1 File — (DOCX) [file pone.0194649.s001.docx]

**Supplemental File 1: Data Dictionary for Study Dataset**

**Urbanicity:** Describes in text whether the cancer center completing the survey is located in an urban area or in a rural/suburban area

**Type_Cancer_Center:** Type of cancer center is based upon Commission of Cancer (CoC) categories for cancer centers. More information about the meaning of each of these categories is available on the CoC website.

Each cancer center is recorded in text as “Academic comprehensive cancer program,” “Comprehensive community cancer program,” “Community cancer program,” Veterans Administration cancer program,” or “Integrated network cancer program”

**Pt_nav:** This variable describes in text the level of availability of patient navigation at each of the cancer centers.

Each cancer center is recorded in text as having patient navigation “Regularly available,” “Available for some patients” or “Not available”

**Blood_Lymph:** This variable describes whether patient navigators are available for patients with cancers of the blood/lymph systems.

1=Yes; 0=No

**Bone_Marrow:** This variable describes whether patient navigators are available for patients with cancers of the bone/blood marrow.

1=Yes; 0=No

**Brain_Spinal:** This variable describes whether patient navigators are available for patients with cancers of the brain/spinal cord.

1=Yes; 0=No

**Breast:** This variable describes whether patient navigators are available for patients with breast cancer.

1=Yes; 0=No

**GI:** This variable describes whether patient navigators are available for patients with gastrointestinal cancers.

1=Yes; 0=No

**GU:** This variable describes whether patient navigators are available for patients with genitourinary cancers.

1=Yes; 0=No

**Gyn:** This variable describes whether patient navigators are available for patients with gynecological cancers.

1=Yes; 0=No

**Head_Neck:** This variable describes whether patient navigators are available for patients with head and neck cancers.

1=Yes; 0=No

**Thoracic:** This variable describes whether patient navigators are available for patients with thoracic cancers.

1=Yes; 0=No

**Skin:** This variable describes whether patient navigators are available for patients with skin cancers.

1=Yes; 0=No

**Ped:** This variable describes whether patient navigators are available for patients with pediatric cancers.

1=Yes; 0=No

**Sarcoma:** This variable describes whether patient navigators are available for patients with sarcoma cancers.

1=Yes; 0=No

**Lay_nav:** This variable indicates that the cancer center uses lay navigators to assist patients.

1=Yes; 0=No

**Prof_nav:** This variable indicates that the cancer center uses professionally prepared navigators (nurses, social workers) to assist patients.

1=Yes; 0=No

**Screening:** This variable indicates that the cancer center has patient navigators available during the process of cancer screening.

Coded as text: “Yes” or “No”

**Diagnostics:** This variable indicates that the cancer center has patient navigators available during the process of cancer diagnostics.

Coded as text: “Yes” or “No”

**Treatment:** This variable indicates that the cancer center has patient navigators available during the process of cancer treatment.

Coded as text: “Yes” or “No”

**Post_TX:** This variable indicates that the cancer center has patient navigators available during the period after completion of primary cancer treatment (i.e. the post-treatment period).

Coded as text: “Yes” or “No”

**Distress_screen:** This variable describes in text which patients at the cancer center receive distress screening services.

Response options are recorded in text as “Routinely conducted,” meaning services are routinely conducted at the cancer center; “Not Routine but Referred as Needed,” meaning services are not routinely conducted but patients are referred as needed for psychological services, or “Not part of routine care” meaning psychosocial assessment and referral is not a part of routine care at the cancer center.

**Initial_visit:** This variable indicates whether the cancer center has distress screening services available for patients at the point of the initial cancer center visit.

Coded as text: “Yes” or “No”

**At_dx:** This variable indicates whether the cancer center has distress screening services available for patients at the point of cancer diagnosis.

Coded as text: “Yes” or “No”

**Start_TX:** This variable indicates whether the cancer center has distress screening services available for patients at the start of cancer treatment.

Coded as text: “Yes” or “No”

**End_TX:** This variable indicates whether the cancer center has distress screening services available for patients at the end of cancer treatment.

Coded as text: “Yes” or “No”

**Long_term_fu:** This variable indicates whether the cancer center has distress screening services available for patients during long term patient follow up (i.e. after completion of primary treatment).

Coded as text: “Yes” or “No”

**Recurrence:** This variable indicates whether the cancer center has distress screening services available for patients at the point of cancer recurrence.

Coded as text: “Yes” or “No”

**Incurability:** This variable indicates whether the cancer center has distress screening services available for patients at the point that they learn that their cancer is not curable.

Coded as text: “Yes” or “No”

**EOL_discuss:** This variable indicates whether the cancer center has distress screening services available for patients at the point that they discuss end of life options with patients.

Coded as text: “Yes” or “No”

**Distress_Screen_Tool:** This variable, recorded in text, indicates what distress screening tools that the cancer center reports using.

Coded as text: “NCCN-Therm”, “POMS,” or “None”

**Care_Plan_dx_info:** This variable indicates the frequency in which the cancer center provides information about diagnosis information in a survivorship care plan to their patients.

Response options are recorded as “Never,” “Rarely,” “Sometimes,” “Very Often,” or “Always.”

**Care_plan_TX:** This variable indicates the frequency in which the cancer center provides information about what treatment the patient received in a survivorship care plan to their patients.

Response options are recorded as “Never,” “Rarely,” “Sometimes,” “Very Often,” or “Always.”

**Care_plan_sched_fu:** This variable indicates the frequency in which the cancer center provides information about the appropriate schedule for follow up visits and tests in a survivorship care plan to their patients.

Response options are recorded as “Never,” “Rarely,” “Sometimes,” “Very Often,” or “Always.”

**Care_plan_late_eff:** This variable indicates the frequency in which the cancer center provides information about potential long term and late effects of cancer and its treatment that the patient should be alert for in a survivorship care plan to their patients.

Response options are recorded as “Never,” “Rarely,” “Sometimes,” “Very Often,” or “Always.”

**Care_plan_lifestyle:** This variable indicates the frequency in which the cancer center provides advice on important lifestyle issues (i.e. physical activity, smoking and diet) in a survivorship care plan to their patients.

Response options are recorded as “Never,” “Rarely,” “Sometimes,” “Very Often,” or “Always.”

**Care_plan_symptoms:** This variable indicates the frequency in which the cancer center provides information about what symptoms the patient should watch for in a survivorship care plan to their patients.

Response options are recorded as “Never,” “Rarely,” “Sometimes,” “Very Often,” or “Always.”

**Care_Plan_supp_res:** This variable indicates the frequency in which the cancer center provides a list of support resources in a survivorship care plan to their patients.

Response options are recorded as “Never,” “Rarely,” “Sometimes,” “Very Often,” or “Always.”

**Cancer_Plan_Tool_Used:** This variable describes in text what survivorship care planning template the cancer center provides to patients.

Response options are recorded in text as “Journey Forward,” “NCCN,” “Star Survivorship,” “Cancer Center developed,” and “None.”

**Surv_staff:** This variable describes whether the cancer center as a designated staff person who is responsible for coordinating survivorship care planning services at the cancer center.

Responses options are recorded in text as “Yes” or “No”

**Gen_risk_assess:** This variable describes to which patients at the cancer center genetic risk assessment is provided.

Response options are recorded in text as “Yes, All,” meaning services are systematically provided across the cancer center, “Yes, Some,” meaning services are not systematically provided across the cancer center; but they are available to some patients; and No” meaning services are not generally provided.

**Gen_couns:** This variable describes to which patients at the cancer center genetic counseling is provided.

Response options are recorded in text as “Yes, All,” meaning services are systematically provided across the cancer center, “Yes, Some,” meaning services are not systematically provided across the cancer center; but they are available to some patients; and No” meaning services are not generally provided.

**Active_pal_program:** This variable describes the availability of a currently active palliative care program at the cancer center.

Response options are recorded in text to indicate that these services are “Available on Site,” “Available by Referral” or Not Available.”

**Onc_pal_MD:** This variable describes the level of availability of at least one palliative care oncologist at the cancer center.

Response options are recorded in text to indicate that these services are “Available on Site,” “Available by Referral” or Not Available.”

**Onc_pal_nurse:** This variable describes the level of availability of at least one palliative care nurse at the cancer center.

Response options are recorded in text to indicate that these services are “Available on Site,” “Available by Referral” or Not Available.”

**Inpt_consult_team:** This variable describes the availability of an inpatient palliative care consultation team at the cancer center.

Response options are recorded in text to indicate that these services are “Available on Site,” “Available by Referral” or Not Available.”

**Outpt_clinic:** This variable describes the availability of a palliative care outpatient clinic at the cancer center.

Response options are recorded in text to indicate that these services are “Available on Site,” “Available by Referral” or Not Available.”

**Pal_beds:** This variable describes the availability of dedicated palliative care beds at the cancer center.

Response options are recorded in text to indicate that these services are “Available on Site,” “Available by Referral” or Not Available.”

**Hospice:** This variable describes the availability of a hospice program at the cancer center.

Response options are recorded in text to indicate that these services are “Available on Site,” “Available by Referral” or Not Available.”
